# Follow-Up of Women with Cervical Cytological Abnormalities: Progression and Regression Events 

**DOI:** 10.31557/APJCP.2019.20.4.1019

**Published:** 2019

**Authors:** Aniúsca Vieira dos Santos, Giovana Tavares dos Santos, Rosicler Luzia Brackmann, João Carlos Prolla, Claudia Giuliano Bica

**Affiliations:** 1 *Pathology Research Laboratory,*; 3 *Department of Basic Health Sciences, Federal University of Health Sciences of Porto Alegre, *; 2 *Laboratory of Pathology, Santa Casa de Misericórdia of Porto Alegre, Rio Grande do Sul, Brazil.*

**Keywords:** Cervical cancer, screening, diagnosis, progression, regression

## Abstract

**Section Title:**

Abnormalities in the cervix, when identified early by Pap smear, can be treated in the early stages or in the precursor stages of the neoplasia, which may increase the chances of regression of the lesion. The aim to verify the rate of cervical abnormalities and to evaluate the risk of progression or regression associated with age and cytological diagnosis.

**Methods:**

The study was conducted in a referral hospital in Southern Brazil, based on the results of pathology and cytopathology laboratory tests of uterine cervix. The historical cohort included patients with an abnormal cytology diagnosis in the period from January 2010 to December 2014, followed until July 2016.

**Results:**

A total of 42,389 cervical smears were analyzed, 4,427 of which were eligible for analysis of the evolution of cervical abnormalities. In progression and regression events analysis, we observed that patients with a cytological diagnosis of atypical glandular cells presented a higher risk of cervical abnormality progression (Hazard Ratio: 2.0 and 95% confidence intervals 1.36–3.48). We also observed that patients younger than 25 years old were more likely to regress the cervical lesions (Hazard Ratio:1.4 and 95% confidence intervals 1.20–1.74).

**Conclusions:**

The associations found between the events (progression and regression), age and cytological diagnosis, highlights the importance of cytological screening in populations at risk of precursor of cervical cancer lesions, especially in women older than 25 years.

## Introduction

Worldwide, cervical cancer is the most common neoplasm of the female genital tract, accounting for approximately 530,000 new cases and 265,000 deaths per year (Ferlay et al., 2015). More than 85% of cases diagnosed with cervical cancer live in underdeveloped or developing countries (Torre et al., 2015). Among these countries, Brazil stands out, having the third place with respect to prevalence of cervical cancer in among women (INCA, 2015).

These high rates are related to the Human Papillomavirus (HPV) infection, as the main risk factor of the disease. HPV infection is one of the most common sexually transmitted infection, and makes cervical cancer a serious public health problem (IARC, 2007; Koshiol et al., 2008; zur Hausen, 2009; de Sanjose et al., 2010). However, there are other factors that may influence the acquisition and persistence of viral infection as well as regression, stabilization or progression of cervical lesions and atypia, causing different rates of disease around the world (zur Hausen, 2002; Castellsagué and Muñoz, 2003; de Freitas et al., 2012; WHO, 2014; CDC, 2018). 

In this regard, screening programs based on cytology examinations continue to be the mainstay of cervical cancer prevention, especially in underdeveloped or developing countries. When identified early, abnormalities in the uterine cervix may be treated in the early stages or in the precursor phases of the neoplasm, increasing the lesion regression chances (WHO, 2014; INCA, 2015).

According to the results of cytological for the diagnosis of cervical cancer, it is possible to update indicators of rates of progression or regression of cervical abnormalities, re-evaluate the effectiveness of health programs, and develop strategies to reduce the incidence and mortality of this disease. The objective of this study was to analyze the frequency, progression, and regression of cervical cancer precursor lesions associated with age and cytological diagnosis in a reference health service in oncology.

## Materials and Methods

*Data*

This study was conducted in a referral hospital for oncology in southern Brazil, with a historical cohort and dynamic population. This population was not included in the public health strategy of vaccination against HPV. 

Through computerized hospital system, we had retrieved information on all cervical cytological examinations, including primary smears of screening programs, opportunistic screening, and secondary tests regardless of whether they were taken under the public system or private healthcare, which was released from January 2010 to December 2014.

In the all abnormal cytology tests was verified corresponding patient of the cervical smear, through coded data . For each patient, the type of cervical lesion and the evolution of abnormalities in the uterine cervix (progression or regression) were analyzed. The evolution of abnormalities was observed during the follow-up period, between January 2010 and July 2016, through cytological and histopathological assessments.

*Study Definitions*

In order to define the progression or regression status of cervical abnormalities, all cervical diagnoses during follow ups were verified, regardless of the treatment strategies. We considered the cases in which, there was a second diagnosis with normal results after the first cytological diagnosis during the follow-up as a regression. Cases in which a second abnormal diagnosis occurred, after the first cytology diagnosis, with a higher degree of cervical damage were considered as cases with progression of the abnormality. 

We categorized the cervical diagnoses according to the Bethesda System for Cervico-vaginal Cytopathology (Solomon et al., 2002) and standardized for Neoplasms. Accordingly, the following abbreviation were used: CA for stands for cervical adenocarcinoma and CC for cervical carcinoma. For Abnormalities precursor of the neoplasm, we applied these abbreviations: AGC for atypical glandular cells, ASC for atypical squamous cells, LSIL for low-grade squamous intraepithelial lesions, and HSIL for high-grade squamous intraepithelial.

*Quality Assurance*

The pathology laboratory of our referral hospital is submitted to some quality assurance procedures. In the cytology unit, all cytological exams are analyzed by two independent cytologists with great expertise, reducing inter-observer variable errors. In addition, every case of abnormal cytology is reviewed daily and 10% of negative cytology cases are randomly reviewed. In addition, an external quality control of the cytological exams linked to the public health system is performed by an External Quality Monitoring Unit (UMEQ), According to recommendations presented by the Brazilian National Cancer Institute José Alencar Gomes da Silva (INCA), the Brazilian Society of Pathology (SBP), and the Brazilian Society of Clinical Cytology (SBCC).

*Statistical analysis*

The incidence rate was calculated for regression and progression of cervical lesions between 2010 and 2016, by age-specific rate and age-standardized per 100,000 person-months (ASR), according to the world female population (UN, 2015). To compare the mean age at the initial cytological diagnosis, the ANOVA test was used and the Tukey’s test was used for multiple comparisons. The Kaplan–Meier curves and the Log-Rank test were run to verify the occurrence time of each event (progression or regression) and compare the levels of the analyzed factors (baseline characteristics of age at event and cytology smear results).

The hazard ratio (HR) of the events was also determined by COX Regression test, depending on the initial cytology diagnosis and the age group of the patient in the event (<25 years, 25–60 years,> 60 years). Crude results (HR) was adjusted for age during the initial study follow-up (aHR) along with conducting multiple analysis in the regression model. The statistical difference of the data was considered significant when p <0.05. Confidence intervals 95% was also considered. All data were compiled in a spreadsheet and analyzed using SPSS (version 23).

*Ethics approvals*

This study was in accordance with the Declaration of Helsinki, the Universal Declaration on Bioethics and Human Rights, and Resolution 466/12 of the National Health Council of Brazil. It was approved by the Research Ethics Committees of the reference institutions (Universidade Federal de Ciências da Saúde de Porto Alegre and Complexo Hospitalar Santa Casa). This article does not contain any studies with animals performed by any of the authors .

## Results

From the total of 42,389 cytological samples screened for cervical cancer and precursor lesions between January 2010 and December 2014, 4,709 (11.1%) abnormal cytology tests (annual average 2.75%) were identified. From these tests, 4,427 abnormal tests with complete data were selected, which corresponded to 3,693 women (with at least one abnormal cytological examination).

**Figure 1:**
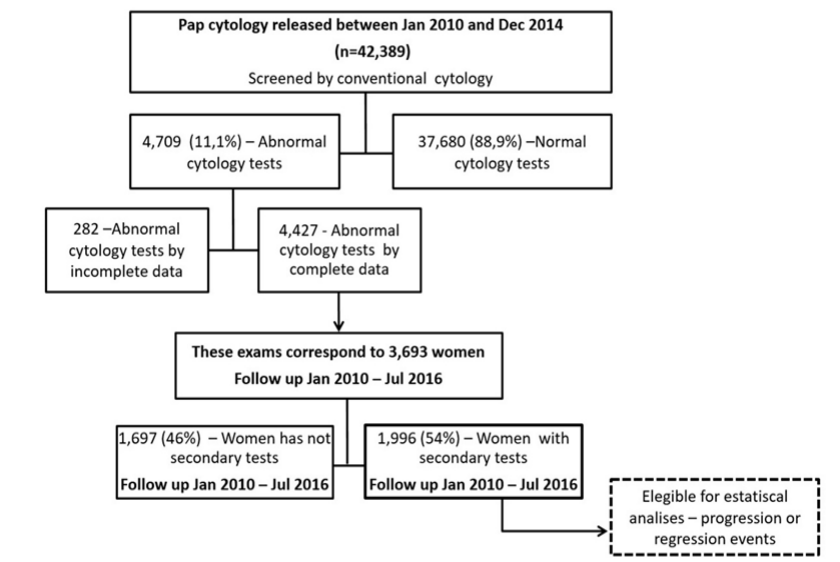
Study Population - Details on Retrospective Cohort

This group of women was eligible as the historical cohort, being followed up by the examinations performed between January 2010 and July 2016. However, in the analysis of individual disease history, only 1,996 (54%) women had follow-up examinations (Figure 1). The analysis of the initial cytological diagnosis of each group and follow-up is shown in Table 1.

**Table 1:** Results of All Cervical Smears and Complete Range of Follow-up Results

Colunas1	N	%
Total women	3,693	100.0
Pap Cytology		
AGC	123	3.3
CA	0	0.0
CC	2	0.1
HSIL	65	1.8
LSIL	570	15.4
ASC	2,933	79.4
Followed up women1	1,996	54.0
Loss of follow-up	1,697	46.0
Follow up results	1,996	100.0
Pap Cytology		
AGC	87	4.4
CA	0	0.0
CC	2	0.1
HSIL	48	2.4
LSIL	303	15.2
ASC	1,556	78.0
Progression or Regression events		
Non Events	17	0.9
Events	1,979	99.1

AGC, atypical glandular cells; CA, cervical adenocarcinoma; CC, cervical carcinoma; HSIL, high-grade squamous intraepithelial lesions; LSIL, low-grade squamous intraepithelial lesions; ASC, atypical squamous cells; 1Period of follow-up between January 2010 and July 2016.

**Figure 2:**
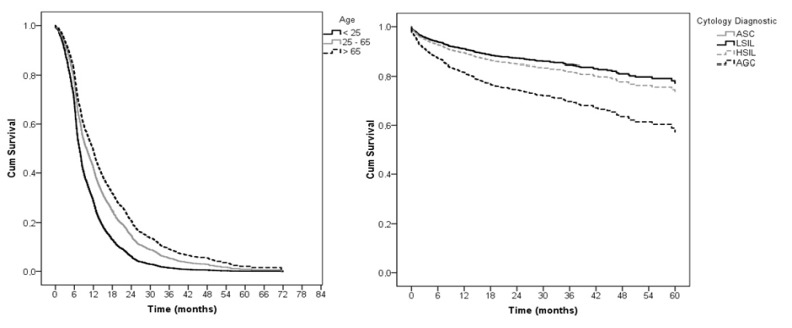
A, Hazard risk of regression event in association with group age woman (ref. age 25-65 years); B, Hazard risk of progression event in association with cytology diagnostic (ref. cytology diagnostic at AGC)

**Table 2:** Regression Rates after Follow-up between 2010 and 2016, According to Age Group and Cytology Diagnosis

	Mean Time (in months)	SE (95% CI)	Univariate HR Crude (95%CI)	p-Value	Bivariate aHR (95%CI)	p-value
Age in years						
<25	12.4	0.7 (11.1 - 13.8)	1.0 (0.9 - 1.2)	0.36	1.4 (1.2 - 1.7)	< 0.01
25-65	13.5	0.3 (12.9 - 14.1)	(ref)		(ref)	
>65	11.4	1.3 (8.8 - 14.0)	1.2 (0.9 - 1.5)	0.13	0.8 (0.6 - 1.1)	0.18
Pap Cytology^2^						
ASC	13.1	0.3 (12.5 - 13.7)	(ref)		(ref)	
LSIL	14.6	0.8 (13.0 - 16.3)	0.8 (0.7 - 1.0)	0.06	1.0 (0.8 - 1.2)	0.87
HSIL	15.1	2.5 (10.0 - 20.2)	0.9 (0.6 - 1.2)	0.61	0.9 (0.7 - 1.2)	0.68
AGC	13.3	1.6 (9.9 - 16.6)	1.0 (0.8 - 1.3)	0.75	0.9 (0.6 - 1.4)	0.83

ASC, atypical squamous cells; LSIL, low-grade squamous intraepithelial lesions; HSIL, high-grade squamous intraepithelial lesions; AGC, atypical glandular cells. SD, standard deviations; SE, standard error; CI, confidence interval; HR, hazard ratio; aHR, Hazard ratio adjusted of age; Ref, Reference variable; 1, ASR, Age-standardized incidence rate per 100.000 person-months. All person-months observation (regression event): 19.859; 2, Only pre-malignant cervical lesions.

**Table 3:** Progression Rates after Follow-up between 2010 and 2016, According to Age Group and Cytology Diagnosis

	Mean Time (in months)	SE (95%CI)	Univariate HR Crude (95%CI)	p value	Bivariate aHR (95%CI)	p value	Multiple analysis aHR (95%CI)	p value
Age in years								
<25	50.3	2.7 (44.9 - 55.7)	1.6 (1.1 - 2.4)	< 0.01	1.3 (0.8 - 2.0)	0.21	1.3 (0.8 - 2.0)	0.19
25-65	62.6	0.7 (61.1 - 64.2)	(ref)		(ref)		(ref)	
>65	57.5	2.7 (52.1 - 62.9)	1.2 (0.7 - 2.3)	0.39	1.8 (0.9 - 3.6)	0.90	1.8 (0.9 - 3.6)	0.09
Pap Cytology^2^								
ASC	62.4	0.8 (60.8 - 64.1)	(ref)		(ref)		(ref)	
LSIL	61.9	1.7 (58.4 - 65.4)	1.1 (0.7 - 1.5)	0.52	1.0 (0.7 - 1.4)	0.93	0.9 (0.6 - 1.3)	0.93
HSIL	54.0	2.9 (48.2 - 59.9)	1.2 (0.5 - 2.8)	0.57	1.2 (0.5 - 2.7)	0.64	1.2 (0.5 - 2.7)	0.62
AGC	49.7	3.3 (43.1 - 56.2)	1.9 (1.2 - 3.1)	< 0.01	2.1 (1.3 - 3.4)	<0.01	2.1 (1.3 - 3.4)	< 0.01

ASC, atypical squamous cells - of undetermined significance or cannot rule out a high grade lesion; LSIL, low-grade squamous intraepithelial lesions; HSIL, high-grade squamous intraepithelial lesions; AGC, Atypical glandular cells. SD, standard deviations; SE, standard error; CI, confidence interval; HR, hazard ratio; aHR, Hazard ratio adjusted of age; Ref, Reference variable; 1ASR, Age-standardized incidence rate per 100.000 person-months. All person-months observation (progression event): 2.704; 2, Only pre-malignant cervical lesions.

The mean age of the patients at the beginning of the follow-up was associated with diagnoses of LSIL and AGC (p <0.05), indicating that group with a cytology diagnosis of atypical glandular cells (AGC) were the oldest in the cohort with a mean age of 48.4 ± 11.0 years old. 

On the other hand, women with a cytological diagnosis of low-grade squamous intra-epithelial lesions (LSIL) were the youngest with a mean age of 33.17 ± 11.9 years. Women with a cytology diagnosis of ASC or high-grade squamous intra-epithelial lesions (HSIL) did not present statistically significant differences in relation to age. 

We observed no statistically significant differences for the regression event in the comparison of the free-event survival between the age group at the time of the event and the pathology variable by the beginning of the study (Table 2). 

However, for the progression event, we found the shortest time to progression of cervical abnormalities (49 months) in women with a cytology diagnosis of AGC . By contrast, the longest time to progression events (62.4 months) was found in women with a cytological diagnosis of ASC (Table 3). 

Following hazard ratio analyses, after adjusting for age at the time of the initial cytological diagnosis (aHR), we found that women aged <25 years old had the risk of 1.4 for regression of cervical abnormalities when compared to women aged 25–65 years old (p <0.01 and 95% CI 1.20–1.74). The hazard ratio for regression did not present statistically significant difference in relation to cytological diagnosis (Table 2).

In terms of progression event, we found that women with a cytological diagnosis of AGC had a 1.9-fold increased risk for progression of cervical abnormalities when compared to women with a cytological diagnosis of ASC (95% CI 1.24–3.13 p <0.01). This found remained after the bivariate and multivariate analyses (Table 3).

The women diagnosed with AGC presented a twofold increased risk of progression of cervical abnormalities in comparison to women with an initial diagnosis of ASC according to bivariate analysis (95% CI 1.36–3.48 and p <0.01). After multiple analysis, 2.1 aHR for progression event was identified for women diagnosed with AGC (95% CI 1.3–3.4 and p <0.01), but no statistically significant difference was found in relation to age (Table 3). Figure 2 shows the survival curves for progression and regression events after age adjustmentand according to baseline characteristics of age and cytological diagnosis.

## Discussion

In our study, a significant association between cytology diagnosis and progression of cervical abnormalities was found. In addition, we discovered that women diagnosed with AGC presented a higher risk of progression of cervical abnormalities compared with women with ASC diagnosis. We also perceived that women with an initial cytology diagnosis of AGC presented the progression event in a shorter period of time than women with a cytology diagnosis of ASC, LSIL, or HSIL. 

The bivariate analysis indicated that the two isolated factors (age group and cytological diagnosis) were related to the progression event. When we performed a multiple analysis in the regression model, we found that only the initial cytological diagnosis of AGC remained statistically significant (aHR 2.1). It is noteworthy to mention that abnormalities in cervical glandular epithelium were relatively uncommon, comprising less than 5% of cervical smear test results in our study. Nevertheless, a strong association was found between initial cytological diagnosis of AGC and the progression event.

Our findings are in accordance with the results of a recent study by Wang et al., (2016), which found that cytological diagnosis of AGC observed in cervical screening was associated with a persistent high risk of cervical cancer for up to 15 years, particularly for cervical adenocarcinoma. Similar results were also reported by Cheng et al., (2011), whereupon women with a first cytological diagnosis of AGC had significantly increased rates of gynecological neoplasms, being 17,85 times more suitable for cancer of the cervix, in comparison to the general screening population.

After adjustment for age in the cytologic diagnosis, we observed a significant association between regression event and , age, in a way that women younger than 25 years experienced a higher regression rate of the abnormalities than women aged between 25 to 65 years (aHR of 1.4). This association between regression of cervical abnormalities and age was also identified in other studies, with a variability in the regression rates of 59.5% to 84% according to the characteristics of the studied population (type of cervical lesion and the specific age group) and the time of follow-up (van Oortmarssen and Habbema, 1991; Morrison et al., 1996; Munro et al., 2016).

The risk of regression evaluation according to the patient’s age has a fundamental role in the elaboration of cytological screening strategies, assisting the definition of the target population. Due to the high regression rate of cervical abnormalities in young patients, studies suggest that screening in women with less than 25 years old has no impact on reducing the incidence or mortality of cervical cancer, and that cytological screening for cervical cancer would be less efficient when compared to cytological screening in older women (Vicus et al., 2014; Munro et al., 2016). 

However, there are still divergences in the recommendations for the age group of cytological screening, especially in countries that use complementary strategies to control cervical cancer such as prophylactic vaccination for HPV subtypes and identification of the virus in the female population, but it is not the reality in underdeveloped or developing countries (INCA, 2015; Lees et al., 2016; Smith et al., 2017). 

Studies on the evolution of cervical abnormalities are fundamental in the epidemiological surveillance of cervical cancer, mainly in underdeveloped or developing countries. The relevance of this study was evident for being one of the most current studies on progression and regression of atypias and precursor lesions of cervical cancer on 42,389 cytological samples screened for cervical cancer, using cytology and pathological data, in a Brazilian hospital reference center.

Our findings on the risk for abnormalities cervical regression and progression according to age group can contribute to provide recommendations for cytology screening tests to diagnose cervical cancer and precursor lesions. Moreover, the identified association between cytology diagnosis of AGC in initial follow-up and the increased risk of progression of cervical abnormality indicated the necessity of conducting further studies to assess other (co) risk factors related to the evolution of abnormalities in cervical glandular epithelium.

## Conflict of interest

The authors declare they have no conflicts of interest.
